# *Wnt-5a *occludes Aβ oligomer-induced depression of glutamatergic transmission in hippocampal neurons

**DOI:** 10.1186/1750-1326-5-3

**Published:** 2010-01-18

**Authors:** Waldo Cerpa, Ginny G Farías, Juan A Godoy, Marco Fuenzalida, Christian Bonansco, Nibaldo C Inestrosa

**Affiliations:** 1 Centro de Envejecimiento y Regeneración (CARE), Centro de Regulación Celular y Patología "Joaquín V. Luco" (CRCP), MIFAB, Departamento de Biología Celular y Molecular, Facultad de Ciencias Biológicas, Pontificia Universidad Católica de Chile, Santiago, Chile; 2 Centro de Neurobiología y Plasticidad del Desarrollo (CNDP), Departamento de Fisiología, Facultad de Ciencias, Universidad de Valparaíso, Valparaíso, Chile

## Abstract

**Background:**

Soluble amyloid-β (Aβ;) oligomers have been recognized to be early and key intermediates in Alzheimer's disease (AD)-related synaptic dysfunction. Aβ oligomers block hippocampal long-term potentiation (LTP) and impair rodent spatial memory. *Wnt *signaling plays an important role in neural development, including synaptic differentiation.

**Results:**

We report here that the *Wnt *signaling activation prevents the synaptic damage triggered by Aβ oligomers. Electrophysiological analysis of Schaffer collaterals-CA1 glutamatergic synaptic transmission in hippocampal slices indicates that *Wnt-5a *increases the amplitude of field excitatory postsynaptic potentials (fEPSP) and both AMPA and NMDA components of the excitatory postsynaptic currents (EPSCs), without modifying the paired pulse facilitation (PPF). Conversely, in the presence of Aβ oligomers the fEPSP and EPSCs amplitude decreased without modification of the PPF, while the postsynaptic scaffold protein (PSD-95) decreased as well. Co-perfusion of hippocampal slices with *Wnt-5a *and Aβ oligomers occludes against the synaptic depression of EPSCs as well as the reduction of PSD-95 clusters induced by Aβ oligomers in neuronal cultures. Taken together these results indicate that *Wnt-5a *and Aβ oligomers inversely modulate postsynaptic components.

**Conclusion:**

These results indicate that post-synaptic damage induced by Aβ oligomers in hippocampal neurons is prevented by non-canonical *Wnt *pathway activation.

## Background

*Wnts *are a family of secreted proteins that bind to Frizzled receptors to activate intracellular signaling cascades, including the *Wnt*/β-catenin [1], *Wnt*/Ca^2+ ^[2] and *Wnt*/planar cell polarity (PCP) pathways [3]. The current classification of the *Wnt *pathways differentiate between the "β-catenin dependent" and "β-catenin independent" pathways. β-catenin independent or non-canonical *Wnt *pathways include the activation of several targets such as Protein Kinase C (PKC), Calcium Calmodulin Kinase 2 (CaMKII) and Jun N-terminal Kinase (JNK). *Wnt-*signaling controls neural patterning and differentiation, including hippocampal formation, dendritic morphogenesis, axon guidance and synapse formation [4,5]. In fact, *Wnt-3a *modulates long-term potentiation (LTP), suggesting a role for *Wnt *signaling in the regulation of synaptic plasticity [6]. Small synthetic molecules mimic *Wnts *leading to both increased spontaneous and evoked neurotransmission that occurs in a transcription-independent fashion [7]. We had previously showed that *Wnt-7a *increases neurotransmitter release modulating the presynaptic component [8]. Deregulation of *Wnt *signaling has been suggested as an etiological cause for specific mental disorders. For example, *Wnt *signaling is upregulated in schizophrenic brains [9] and β-catenin levels were markedly reduced in Alzheimer's disease (AD) patients carrying autosomal dominant PS-1 inherited mutations [10]. The amyloid-β-peptide (Aβ) has been shown to decrease β-catenin levels in cultured neurons, interfering with normal *Wnt *signaling [11,12].

In the amyloid cascade hypothesis of AD, Aβ neurotoxicity has its origin in the binding of Aβ oligomers to the post-synaptic region [13], or affecting vesicular transmitter release [14-16]. Patients in the early stages of AD present synaptic alterations [13,17], without clear neuronal loss. Transgenic (Tg) mice with familial AD mutations display disruptions of LTP that occur before deposition of Aβ plaques [18,19] and it is a sensitive marker for early AD dysfunction. Evidence obtained in neuronal cell cultures have shown that Aβ directly affect synaptic components including post synaptic protein 95 (PSD-95) [20,21]. PSD-95 is a scaffold protein that interacts directly with N-methyl-D-aspartic acid receptors (NMDARs), modulating their channel properties [22], posttranslational processing [23] and stabilization at the synapses [24]. Additionally, Snyder et al., [25] have shown that the effect of Aβ on endocytosis of NMDARs is likely to contribute to the synaptic dysfunction observed in AD [26]. Other studies have shown that PSD-95 interacts indirectly with α-amino-3-hydroxy-5-methyl-4-isoxazolepropionic acid receptors (AMPARs) through the transmembrane protein stargazin [27] and regulates the trafficking and localization of AMPARs at synapses [28]. We report here that *Wnt-5a *modulates synaptic transmission by a postsynaptic mechanism, which eventually is able to prevent the Aβ synaptotoxicity triggered by the Aβ oligomers.

## Results

### *Wnt-5a *increases synaptic amplitude of glutamatergic transmission without affecting paired pulse facilitation in hippocampal slices

**Figure 1:**
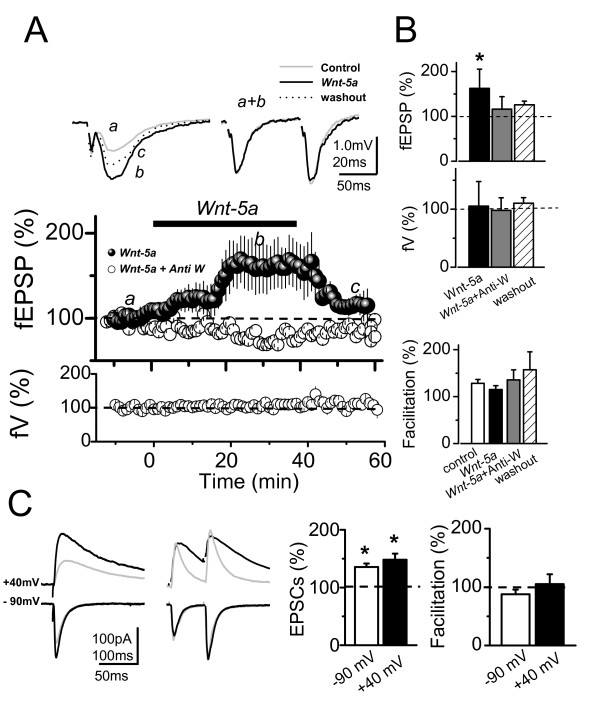
**Field Potential and Intracellular recording of CA1 pyramidal neurons after *Wnt-5a *treatment.** (A, left), Superimposed, average (10 sweeps) of field potentials (fEPSPs) recorded from stratum radiatum of CA1 region before (*a*), after 30 min of *Wnt-5a *perfusion (*b*) and 20 min of washout (*c*). (A, right), Superimposed, normalized (to R1), averaged (10 sweeps) fEPSPs evoked by paired pulse stimulation (100 ms delay) in baseline and in presence of *Wnt-5a *(*a+b*). (A, middle), Time course of effect of *Wnt-5a *(black burbles) or *Wnt-5a *plus a*nti-Wnt-5a *(white burbles) on fEPSPs peak amplitudes. Bottom, effect of *Wnt-5a *plus a*nti-Wnt-5a *on fV. (B), Normalized amplitude of fEPSPs, evoked by the first stimulus (top), in control and after *Wnt-5a *or *Wnt-5a *plus a*nti-Wnt-5a *treatment after 50 min of continued perfusion. Average values of normalized amplitude of fiber volley are shown (center), measured before (control) and after *Wnt-5a *(n = 6) or *Wnt-5a *plus *Anti-Wnt-5a *(n = 4) treatment application. Index of facilitation in both conditions (botton). (C, left), Superimposed, average EPSCs (20 sweeps) evoked by single stimulus and paired pulse stimulation at -90 and +40 mV of holding potential in control conditions (gray trace) and in the presence of *Wnt-5a *(black trace), respectively. (C, right) Summary data of average, normalized EPSCs amplitude and facilitation index, obtained in C in baseline and in the presence of *Wnt-5a *(n = 6), respectively. Bar represents the mean ± SEM (*p < 0.05 Student's t test).

To examine the effects of *Wnt-5a *on excitatory glutamatergic transmission evoked by stimulation of Schaffer collaterals (SC) in hippocampal slices, we recorded the field excitatory postsynaptic potentials (fEPSP) and the excitatory postsynaptic currents (EPSCs) in the presence of 10 μM picrotoxin (PTX) to block GABA_A_-mediated inhibitory synaptic transmission. The fEPSP amplitude increased after 20 min of *Wnt-5a *addition to Artificial CerebroSpinal Fluid (ACSF) perfusion media, 57.5% ± 15.5 (p < 0.05; n = 6), without changing either the fiber volley (fV) amplitude or paired pulse facilitation (PPF) (Figure 1A and 1B). This effect was antagonized completely in the presence of anti-*Wnt-5a*, a generic antibody again *Wnt-5a *domain [29], moreover, this *Wnt-5a *effect was reversible after 20 min of washout (Figure 1A and upper graphs)

To examine the contributions of non-NMDARs (i.e.: AMPARs) and NMDARs in the *Wnt-5a *potentiation of glutamatergic transmission, we recorded intracellulary the EPSCs at different holding potentials in identified CA1 pyramidal neurons (Figure 1C). In these experiments, EPSCs evoked by the same stimulations started immediately after entering whole-cell configuration while the membrane potential (Vm) was held at -70 mV. In order to reduce the contribution of postsynaptically mediated plastic phenomena to the observed effects [30], the EPSCs were obtained 20 min later while briefly clampling the cell at both -90 and +40 mV (< 2 min). The perfusion of *Wnt-5a *increased the EPSCs amplitude evoked at both values of Vm. The EPSCs mean values of AMPARs and NMDARs components, measured at peak and 200 ms after the stimuli respectively, showed an increase of 35% at -90 mV and 48% at +40 mV with respect to control ACSF (Figure 1C and left graph). Under these conditions, the mean values of the PPF did not change in the presence of *Wnt-5a *at both holding potentials (Figure 1C and right graph). These results indicate that the potentiation induced by *Wnt-5a *is due to postsynaptic modulation of the glutamatergic postsynaptic currents mediated by activation of both NMDA and AMPA receptors.

### Aβ oligomers reduce the amplitude of synaptic response without affecting the PPF

**Figure 2:**
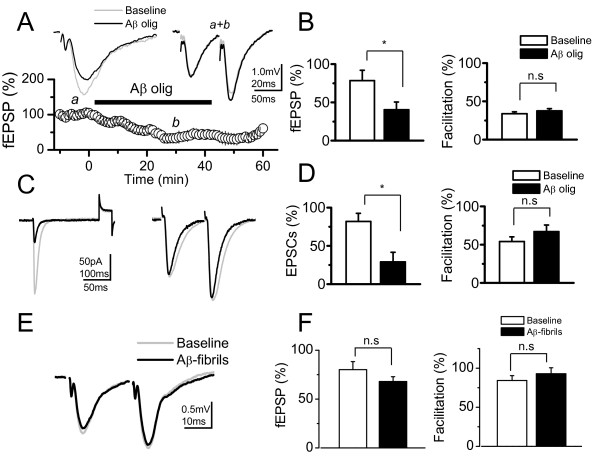
**Aβ oligomers but not Aβ fibrils reduce the amplitude of fEPSP without affect the PPF.** (A, left), Superimposed, average (10 sweeps) of fEPSPs before (*a*) and after 40 min of Aβ oligomers perfusion (*b*). (A, right), Superimposed, normalized (to R1), averaged (10 sweeps) fEPSPs evoked by paired pulse stimulation (100 ms delay) in control and in presence of Aβ oligomers (*a+b*), and time course of Aβ oligomer effects on fEPSPs. a) baseline, b) effect of Aβ oligomers and c) washout with ACSF. (B) Summary data of fEPSPs amplitude (left) and facilitation index in control and after Aβ oligomers treatment. (n = 5). (C) Superimposed, average (10 sweeps) of EPSCs evoked by single and paired pulse protocol, before (control, gray trace) and after 40 min of continued perfusion with Aβ oligomers (black trace). (D) Summary date of EPSCs amplitude (right) and facilitation index (left) in control and after Aβ oligomers treatment, respectively. (n = 5). (E) Superimposed, normalized (to R1), averaged (10 sweeps) fEPSPs evoked by paired pulse stimulation in control and in presence of Aβ fibrils. (F), Summary date of fEPSPs amplitude (left) and facilitation index (right) in control and after Aβ fibrils treatment (n = 4). Bar represents the mean ± SEM (*p < 0.05 Student's t test).

The current hypothesis on AD suggests that the neurotoxic Aβ specifically affects central synapses, in the form of Aβ oligomers [13,20]. Aβ oligomers associate with regions enriched in PSD-95 [20] and reduce the PSD-95 content in both hippocampal neurons [21] and APP transgenic animals [26]. We confirmed such studies and asked whether the *Wnt-5a *activation of the non-canonical pathway is able to overcome the neurotoxic effect of Aβ oligomers. To examine the effects of Aβ oligomers on excitatory glutamatergic transmission in the CA3-CA1 synapses, we recorded fEPSP. The fEPSP amplitude decreased after 20 min of adding 500 nM Aβ to the ACSF perfusion media (Figure 2A), without changing either the fV amplitude (data non-shown) or PPF (Figure 2A). This effect was partially reversible after 20 min of washout (Figure 2A). On average, Aβ induces a decrease of 47% ± 10 of fEPSP amplitude (p < 0.05; n = 5), (Figure 2B, left graph), without affecting facilitation. In fact, mean values of the PPF did not change significantly with respect to their controls in any of these conditions (Figure 2B, right graph). The hippocampal slices treated with Aβ fibrils (500 nM) showed no effects on either the amplitude or facilitation (Figure 2E and 2F). Additional file 1A shows an additional control, using reverse peptide Aβ_42-1 _amplitude of fEPSP. No changes with respect to the baseline were observed.

The intracellular recording experiments carried out using the same condition as that the field potentials confirmed that Aβ oligomers affect the amplitude of the response without affecting the facilitation index. The effect of Aβ oligomers on EPSCs and PPF are showed before (baseline) and 40 min after the application of the Aβ oligomers (Figure 2C). The normalized amplitude of the response was 60% less after of application of Aβ oligomers compared to baseline (Figure 2D, left graph). The mean facilitation index values of do not change in the presence of Aβ oligomers (Figure 2D, right graph). No changes in the PPF indicate that the probability of neurotransmitter release does not change in presence of Aβ and thus that synaptic depression could be due to postsynaptic mechanisms.

### Aβ oligomers reduce both NMDA and AMPA postsynaptic currents

**Figure 3:**
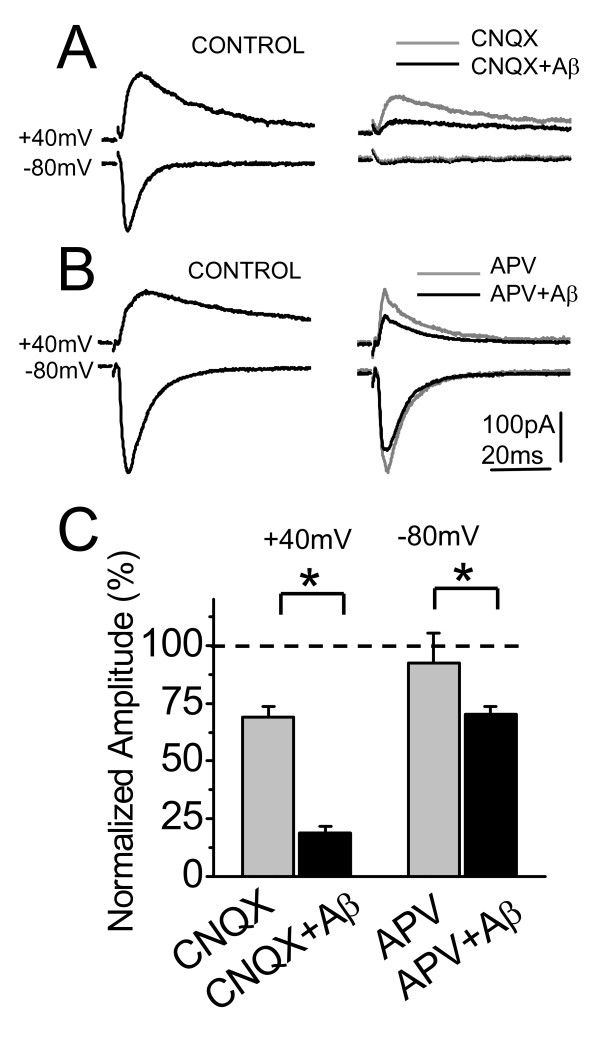
**Aβ oligomers decrease mainly the NMDA current**. (A) Average (20 sweeps) EPSCs in control (left), after treatment with CNQX (gray trace) or CNQX plus Aβ oligomers (black trace) (right) at -80 and +40 mV of holding potential obtained from a same neuron. B) Average (20 sweeps) EPSCs in control (left), after treatment with APV (gray trace) or APV plus Aβ oligomers (black trace) (right) at -80 and +40 mV of holding potential. C) Summary data of normalized amplitude (to control) of evoked EPSCs obtained from experiments on A (n = 6) and B (n = 5). Bar represents the mean ± SEM (*p < 0.05 Student's t test).

Previous evidence has shown that removal of AMPARs is necessary and sufficient to induce the Aβ-mediated synaptic depression [31]. To test whether NMDARs and AMPARs showed different sensitivities to Aβ oligomers, we compared their effects on SC (Shaffer Collaterals) evoked current by clampling the neuron at both +40 and -80 mV, in the presence of the glutamate receptor antagonists DL-2-amino-5-phosphonovaleric acid (50 μM APV) and 6-cyano-7-nitroquinoxaline-2,3-dione (20 μM CNQX), respectively. In the presence of CNQX, the EPSCs at -80 mV were completely abolished, while at +40 mV were reduced until a 65% respect to control ASCF (Figure 3A). Under these conditions, perfusion of 500 nM Aβ oligomers decreased the NMDARs-mediated-response, reaching only 27% of the control EPSC ASCF amplitude. In the presence of APV, the EPSCs amplitude and decay constants (data non shown), measured at both -80 and +40 mV holding potentials were diminished with respect to control ASCF (Figure 3B). The addition of Aβ oligomers reduced the AMPARs-mediated-response reaching only 75% of the EPSC control ASCF amplitude. On average, in the presence of Aβ oligomers the mean values of EPSCs responses mediated by NMDARs (i.e.: CNQX and +40 mV; n = 6) were significantly lower than AMPARs (i.e.: APV and -80 mV; n = 5 neurons) (Figure 3C). The above results show that the synaptic depression induced by Aβ oligomers affects the NMDAR more than the AMPAR receptors.

### *Wnt-5a *occludes the depression induced by Aβ Oligomers on Synaptic Transmission

**Figure 4:**
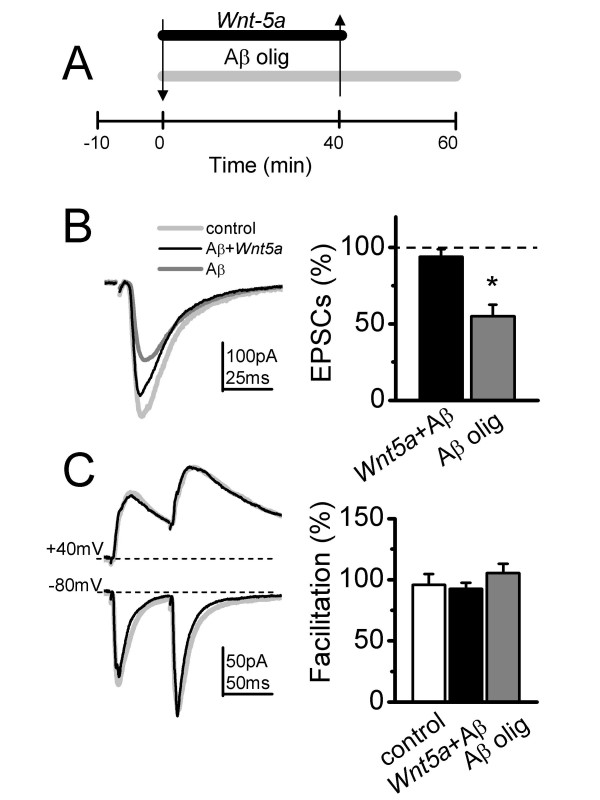
***Wnt-5a *occludes the synaptic transmission depression induce by Aβ oligomers**. (A), Time course of the treatment with Aβ plus *Wnt-5a *(first arrow) and to extra treatment with Aβ oligomers (second arrow). (B), Average (20 sweeps) EPSCs in control (light gray), after 40 min of treatment with Aβ plus *Wnt-5a *treatment (black) and after to extra treatment with Aβ oligomers (dark gray), recorded at -80 mV holding potential. Mean of normalized amplitude of evoked EPSCs in control, Aβ and Aβ plus *Wnt-5a *treated slices (right) (n = 6). (C), Superimposed, average EPSCs evoked by paired pulse test at +40 and -80 mV of holding potential (left). Mean of facilitation index measured before (control) and after *Wnt-5a *plus Aβ or Aβ alone treatment application (right). Bar represents the mean ± SEM (*p < 0.05 Student's t test).

The evidence shows that *Wnt-5a *augments the glutamatergic transmission mainly by a postsynaptic mechanism increasing both NMDA and AMPA currents. Conversely, Aβ oligomers impair synaptic transmission mainly by decreasing the NMDA currents and to a smaller degree the AMPA currents. Therefore, in order to examine whether *Wnt-5a *treatment prevents Aβ oligomer induced synaptic impairment, we treated the hippocampal slices with *Wnt-5a *in the presence of Aβ oligomers (Figure 4A, first arrow), and later with Aβ oligomers alone (Figure 4A, second arrow). Under these conditions, *Wnt-5a *showed protective effects against Aβ oligomers, without changing the EPSCs amplitude. In the same set of experiments, in the presence of Aβ oligomers alone, the removal of *Wnt-5a *from the perfusion media decreased the EPSCs amplitude around 60% at a holding potential of -80 mV (Figure 4B and 4C). In both conditions, the facilitation index did not show any change compared to control, confirming that it is the postsynaptic site where the effects of both peptides take place (Figure 4C). Additionally, we carried out experiments adding Aβ oligomers alone, waiting 30 min to observe the effect, and then allowing that *Wnt-5a *recovers the levels of amplitude of fEPSP (Additional file 1B).

**Figure 5:**
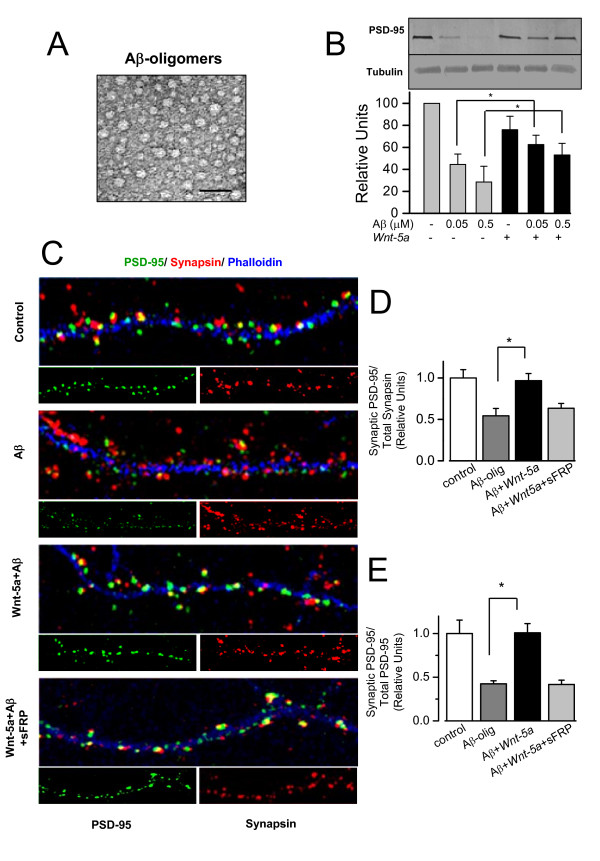
***Wnt-5a *prevents the changes induced by Aβ oligomers on PSD-95 clustering and in synaptic contact.** (A) Electron micrograph of the material corresponding to Aβ-oligomers taken in Phillips Tecnai 12 EM. The picture shows the amylospheroids. Scale bar = 100 nm. (B) Representative immunoblot of total PSD-95 levels of neurons exposed to 0.05 or 0.5 μM Aβ oligomers in the presence or absence of *Wnt-5a *and a quantification of densitometry are shown (n = 3). *P < 0.05. (C), Representative neurite images of double immunofluorescence of PSD-95 (green) and synapsin-1 (red) and stained with phalloidin (blue) from samples subjected for 1 h to control, Aβ, Aβ/*Wnt-5a *and Aβ/*Wnt-5a*/sFRP treatments. Merged images show the apposition of the pre-synaptic (red) and post-synaptic (green) boutons. (D), Quantification of synaptic PSD-95 over total synapsin-1 and; (F), quantification of synaptic PSD-95 over total PSD-95 upon the different treatments used in C (n = 4). Bar represents the mean ± SEM (*p < 0.05 Student's t test).

Aβ oligomers reduce the surface expression of glutamate receptors [25], hence, we examined whether the activation of the non-canonical *Wnt-5a *signaling was able to protect PSD-95 from the synaptotoxic effects of Aβ oligomers. When hippocampal neuron cultures were exposed to Aβ oligomers, a major decrease in PSD-95 clusters was observed. However, co- incubation of Aβ oligomers with *Wnt-5a *showed that the distribution of the PSD-95 was similar to control neurons (Table 1). Co-treatment with the *Wnt *antagonist soluble frizzled receptor protein (sFRP-1) abolished the increase of PSD-95 triggered by *Wnt-5a *(Table 1). Figure 5A shows the species of Aβ oligomer used in this study which corresponds to Aβ oligomers [32]. We evaluated by Western blots the total level of PSD-95 protein. Aβ oligomers decrease PSD-95 levels, while the co-incubation with *Wnt-5a *prevented such changes (Fig. 5B). Additionally, we determined that Aβ oligomers do not affect the levels of *Wnt-5a *in 15DIV neurons, but do decrease neuronal ramifications, as measured MAP-2 staining. However in these ramifications the levels of *Wnt-5a *did not change (Additional file 2A). In the additional file 2B, we determined the levels of *Wnt-5a *in cellular medium, and in conditioned medium used in the treatments.

**Table 1:** *Wnt-5a *ligand induces the clustering of PSD-95, effect of Aβ oligomers: Immunofluoresce in mature hippocampal neurons of 21DIV, every treatment were by 1 h.

Treatments	PSD-95 (Number of clusters in 100 μM of neurite)
Control	24 ± 3
*Wnt-5a*	46 ± 8*
*Wnt-5a*/sFRP	25 ± 4
Aβ	9 ± 3*
Aβ/*Wnt-5a*	23 ± 2
Aβ/*Wnt-5a*/sFRP	11 ± 2*

Aβ 1 μM, (n = 3). *P < 0.01 respect to the control

**Figure 6:**
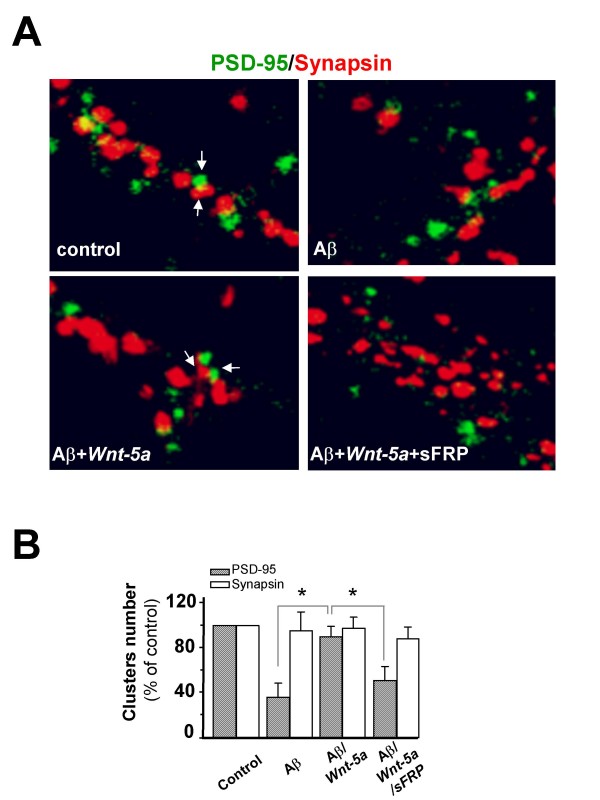
*Wnt-5a *prevents the changes induced by Aβ oligomers in the synaptic contact.(A), Representative neurite images of double immunofluorescence of PSD-95 (green) and synapsin-1 (red), from samples subjected for 1 h to control, Aβ, Aβ/*Wnt-5a *and Aβ/*Wnt-5a*/sFRP treatments. Merged images show the apposition of the pre-synaptic (red) and post-synaptic (green) boutons. (B), Quantification of the number of clausters of the figure A (n = 3). Bar represents the mean ± SEM (*p < 0.05 Student's t test).

Moreover, we evaluated whether *Wnt-5a *prevents the loss of synaptic contacts induced by Aβ oligomer treatment. We quantified the number of synaptic PSD-95 opposite to total synapsin-1 clusters and found that Aβ oligomers decreased the contact between pre-synaptic and post-synaptic regions by almost 55% (Figure 5C), more details in figure 6A. The quantification of PSD-95 and Synapsin is showed in figure 6B. Hippocampal neurons treated with Aβ oligomers in the presence of the *Wnt-5a *ligand did not show any loss of synaptic contacts. The total levels of synapsin did not change (Additional file 3A). In fact, an increase of almost 40% with respect to the effect observed for the Aβ treatment alone was observed in the presence of the *Wnt-5a *ligand (Figure 5C), and this effect was abolished by co-treatment with the *Wnt *antagonist sFRP (Figure 5C). On the other hand, we quantified synaptic PSD-95 in relation to total PSD-95 cluster number. The results indicate that Aβ oligomers decrease the interaction between the pre- and post-synaptic regions, since few PSD-95 clusters remained following Aβ treatment and around 40-50% reduction in the PSD-95-synapsin interaction with respect to the total PSD-95 were observed. *Wnt-5a *prevents the effect triggered by Aβ oligomers at the level of the synaptic structure (Figure 5D and 5E), and the *Wnt *antagonist sFRP abolished the neuroprotective effect of *Wnt-5a *(Figure 5D and 5E). These results show that the changes in the scaffold protein PSD-95 levels induced by the Aβ oligomers are prevented by the activation of the non-canonical *Wnt-5a *signaling pathway.

## Discussion

*Wnt *signaling is essential for neuronal development and the maintenance of the nervous system [12,33-35], including hippocampal formation, dendritic morphogenesis, axon guidance and synapse formation [4,5]. When the neuronal adult circuits are formed *Wnt *probably plays a maintenance role in synaptic connectivity. Synaptic activity cause releases of *Wnt-3a *from synapses and modulates LTP, while inhibition of *Wnt *signaling impairs LTP, and activation of *Wnt *signaling potentiates LTP [6], revealing a synaptic role for *Wnt *signaling in the regulation of the synaptic efficacy in the neuronal adult circuit. Additional evidence shows that modulation of *Wnt *signaling, by a canonical *Wnt-3a *ligand, results in acute enhancement of excitatory transmission in the hippocampus, through a mechanism that might not necessarily involve the transcriptional activity of μ-catenin complexes in the adult CNS [7]. The same authors show that small molecule modulators increase LTP, controlling excitatory transmission. Here we showed that *Wnt-5a *enhances the excitatory transmission. Both fEPSP and EPSCs amplitude, increase in response to *Wnt-5a *treatment in the CA3-CA1 hippocampal circuit. This effect is mainly due to the NMDA current present in the hippocampal slices. For this reason, and knowing the possible effect of Aβ oligomers in NMDA transmission [36,37], we analyzed the possible protective effect of *Wnt-5a *against the Aβ oligomer induced synaptic damage. The synaptic damage induced by Aβ oligomer is considered central in the Alzheimer synaptic failure hypothesis. This hypothesis supports the idea that Aβ causes "synaptic failure" before plaques develop and neuronal death occurs [13]. An important decrease has been demonstrated in the immunoreactivity of the protein synapsin-1 [38] which it has also been observed in AD patients, in which synaptophysin levels are diminished from the early stages of the disease [39]. Furthermore, neurons from APP transgenic mice exhibit decreased PSD-95 levels as well as dendritic spine loss [18,26]. Our electrophysiological experiments show that Aβ oligomer treatments decrease synaptic efficacy, which can be explained by the decreased levels of the PSD-95 observed in hippocampal neuron cultures. We characterize this process by measuring the effect of acute Aβ oligomers application on fEPSPs. This effect was reversible after washing the Aβ oligomers and did not affect the presynaptic machinery of neurotransmitter release. These results suggest that Aβ oligomers mainly affect the postsynaptic region [40]. Others studies have shown that this synaptic damage is produced because Aβ oligomers could affect synaptic components including the PSD-95, by a mechanism that involve the proteasome pathway [20,21]. This decrease in PSD-95 has been related to a reduction in the levels of the GluR1 subunit of the AMPA-glutamate receptors in primary cultures of APP mutant neurons, compared with neurons of wild-type mice [26]. Another report showed that Aβ formation and secretion can be controlled by neuronal activity and secreted Aβ might depresses excitatory synaptic transmission in a NMDA receptor dependent activity [36,37]. Interestingly, EPSCs mediated by NMDARs in slices treated with Aβ oligomers were more affected than the EPSCs mediated by AMPARs, recorded in presence of CNQX and APV, respectively (Figure 3). These findings suggest that Aβ oligomers promote the endocytosis of NMDA and AMPA channels, as it has been reported in hippocampal [31] and cortical neurons [25]. Also, Aβ oligomers diminished the NMDA current and decreased the CREB transcriptional factor required for LTP, memory and lifespan [25]. In the synaptic context, it was recently described that the cellular prion protein (PrP(C)) is a mediator of Aβ-oligomer-induced synaptic dysfunction, because Aβ oligomers bind with nanomolar affinity to PrP(C). Synaptic responsiveness in hippocampal slices from young adult PrP null mice is normal, but the Aβ oligomer blockade of LTP is absent. Anti-PrP antibodies prevent Aβ-oligomer binding to PrP(C) and rescue synaptic plasticity in hippocampal slices from oligomeric Aβ [41].

The impairment produce by Aβ mainly affects the post-synaptic site, including a decrease in PSD-95 levels [20] and glutamatergic channels (AMPARs [26] and NMDARs [36,37]). In our studies *Wnt-5a *modulates the synaptic transmission by a post-synaptic mechanism, indicating that the activation of the non-canonical *Wnt *pathway might protect from the Aβ synaptic damage. Downstream of the *Wnt *ligand exists several options to activate the non-canonical pathways. These include the activation of Frizzled and Dvl which in turn can activate different kinases including PKC, CaMKII and JNK. Concerning the last target in the pathway, JNK, our laboratory recently described that *Wnt-5a*/JNK pathway modulates the post-synaptic region of mammalian synapse directing the clustering and distribution of the physiologically relevant scaffold protein, PSD-95 [42]. A second option of activation might involves the new *Wnt *receptor Ror2 [43,44]. The activation of PKC could directly modulate the phosphorylation of the NR1 NMDA subunits and induce its localization at the synaptic membrane [45]. Other options include the modulation of CaMKII activity and the incorporation of NMDARs in the synapse. Also the activation of JNK could modulate the actin cytoskeleton and produce a remodeling in the dendritic spines structures [46]. All these options imply that *Wnt *is preparing the synapses for defense against possible injury and probably its effect on synaptic, PSD-95 is the most important factor player modulated by *Wnt *in controlling the Aβ damage. We reasoned that when the Aβ treatment occurs in the hippocampal slices in the presence of *Wnt-5a *synaptic impairment is prevented.

## Conclusion

These results suggest that the *Wnt-5a *plays a pivotal role in the maintenance of normal postsynaptic integrity, and its activation may be of therapeutic interest in patients with neurodegenerative diseases such as AD.

## Methods

### Reagents

Synthetic Aβ_1-40 _peptide corresponding to the human Aβ wild-type sequence and the Aβ_1-42 _artic variant [32] were obtained from Chiron Corp. Inc., (Emeryville, CA) and Calbiochem (Postfach, Germany). Antibodies for Synaptic Proteins from Santa Cruz Biotechnology Inc. Immunostaining was also carried out using polyclonal anti-PSD-95, Synapsin-1 and secondary antibody labelled with ^488^Alexa, ^543^Alexa or ^633^Alexa (Affinity Bio Reagents Inc., Golden, CO). To study neuronal morphology phalloidin labelled with TRITC from Molecular Probes (Leiden, The Netherlands) was used.

### Wnt constructs

The different HA-Wnt or sFRP-1 constructs were a kind gift of several individuals, which really made this work possible. *Wnt-5a *was a gift of Dr. Randall T. Moon, University of Washington, Seattle, WA; and sFRP-1 was a gift of Dr. Jeremy Nathans, Johns Hopkins University School of Medicine, Baltimore, MD.

### Cell line culture

Human embryonic kidney 293 cells (HEK-293) were maintained in DMEM supplemented with 10% fetal calf serum (Gibco BRL, Rockville, MD), and 100 ug/ml streptomycin and 100 U/ml penicillin.

### Conditioned medium containing Wnt ligands

*Wnt *ligands were generated in HEK-293 cells transiently transfected by calcium phosphate precipitation [47] with constant and equal amounts of empty vector pcDNA or pcDNA containing the sequences encoding *Wnt-5a *constructs. *Wnt*-conditioned or control media or media containing sFRP-1 were prepared as described [11,48]. *Wnt *secretion was verified by Western blot using an anti-HA antibody (Upstate Biotechnology, Lake Placid, NY) (additional file 3B).

### Primary Rat embryo hippocampal neuron cultures and treatments

Rat primary hippocampal neurons were prepared as previously described [8,11,49]. Hippocampal neurons were obtained from 14 to 21-day-old Sprague-Dawley rat embryos. On day 3 of culture, hippocampal neurons were treated with 1 μM 1-β-D-arabinofuranosylcytosine for 24 h in order to reduce the number of proliferating non-neuronal cells.

### Aβ Oligomers Preparation and Electron Microscopy

The artic Aβ_1-42 _peptide [32] was dissolved in anhydrous and sterile DMSO at 15 mg/ml concentration. For oligomer formation, one aliquot was dissolved in 0.5% PBS at 50 mM final concentration. The sample was subjected to a basic shock adding 2N NaOH to reach pH 12. Then, the sample was neutralized with 1N HCl. The mixture was incubated at room temperature under constant agitation during 1 h to obtain the Aβ oligomers. To visualize Aβ oligomers by electron microscopy, samples were treated as described before [50], and observed using a Phillips Tecnai 12 electron microscope.

### Western Blot

Total protein was prepared from primary rat hippocampal neurons lysed in a buffer RIPA (50 mM Tris-Cl, 150 mM NaCl, 1% NP-40, 0.5% sodium deoxycholate, and 0.1% SDS) supplemented with a protease inhibitor mixture. Equal amounts of protein were resolved using SDS-PAGE, proteins were transferred to PVDF membranes, and immunoblots using anti- PSD-95 and anti-tubulin (Sta Cruz, Biotec. Inc) antibodies.

### Immunohistochemistry

Hippocampal neurons were subjected to different treatments while on coverslips within 24-well plates at a plating density of 30,000 cells/coverslip, fixed with 4% paraformaldehyde/4% sucrose in PBS for 20 min, permeabilized with 0.2% Triton X-100 for 5 min, blocked with 0.2% gelatin and stained with PSD-95 and Synapsin-1 antibodies. Phalloidin coupled to Alexa 633 was used as neurite marker. Digital images of neurons on coverslips were captured with a Zeiss confocal microscope. Images used for quantification were taken with identical microscope settings and analyzed using Image J software (NIH).

### Double antibody sandwich ELISA Techniques

96 wells plates were coated with anti-*Wnt-5a *antibody (Sta Cruz, Biotec. Inc) first antibody antigen capture, the conditioned media the different cultures was concentrated with Amicon tubes and incubated for 1 hr at 37°C. The ligand detection was made with a second monoclonal antibody (R&D Sistem) against *Wnt-5a *ligand. The detection of reaction was made with ABC KIT (Vectastain System, Vector Laboratories, CA. USA) and OPD as substrate.

### Slice preparation and Electrophysiology

Hippocampal slices were prepared essencially as described previously [8]. Then, slices were transferred to an experimental chamber (2 ml), superfused (3 ml/min, at 22-26°C) with gassed ACSF. The experiments were carried out at room temperature (21°C-22°C), measured at the recording chamber. Two recording methods were used: patch clamp [51] and extracellular field potentials recording [52]. *Single cell recording *were made in the whole-cell configuration with fire-polished pipettes (3-5 MΩ) filled with intracellular solution (see below), connected to a tight seal (>1 GΩ). Whole-cell recordings were obtained from the cell body of neurons in the CA1 pyramidal layer. Patch electrodes were made from borosilicate glass and had a resistance of 2-5 MΩ when filled with (in mM); 97.5 K-Gluconate, 32.5 KCl, 10.0 4-(2-hydroxyethyl)-1-piperazine-ethanesulfonic acid (HEPES), 1.0 MgCl2, 5.0 ethyenebis-(oxonitrilo) tetracetate (EGTA) and 4.0 sodium salt (Na-ATP); pH 7.2 (289 mOsm). Neurons were voltage clamped with an EPC-7 amplifier (Heka Instruments), and the experiments started after a 5-10 min stabilization period after access to the intracellular compartment with patch electrodes. The access resistance (10-25 MΩ) was monitored and cells were rejected if it changed more than 20% during the experiment. *Extracellular field potentials recording *[53] were made with a glass pipettes (2-4 MΩ, filled with the perfusion medium), connected to an A.C. amplifier (P-5 Series, Grass), with gain 10000×, LP filter 3.0 kHz and HP filter 0.30 Hz, that was placed in the middle of stratum radiatum of CA1, exactly as described before [8]. The PPF index was calculated by ((R2-R1)/R1), were R1 and R2 are the peak amplitudes of the first and second EPSCs, respectively. Recordings were filtered at 2.0-3.0 kHz, sampled at 4.0 kHz using an A/D converter (ITC-16, Intrutech), and stored with Pulse FIT software (Heka Instruments).

### Statistical analysis

Data were expressed as the mean ± SEM of the values from the number of experiments as indicated in the corresponding figures. Data were evaluated statistically by using the Student's t-test, with P < 0.05 considered significant. ANOVA test was used to compare n differences between experiments.

## Competing interests

The authors declare that they have no competing interests.

## Authors' contributions

WC participated in the design of the experiments, carried out electrophysiological experiments, carried out the interpretation of the results and wrote the manuscript. JGF and JAG participated in cell culture experiments including western blot and immunodetection experiments. MF revised the manuscript. CB participated in the design of the experiments revise the manuscript. NCI design the studies and revise the manuscript. All authors read and approved the final manuscript.

## Supplementary Material

Additional file 1 **(A), Time course of effect of Aβ_42-1 _(gray circles) on fEPSPs peak amplitudes (n = 4).** (B), Time course of effect of Aβ oligomers and *Wnt-5a *after (gray circles) on fEPSPs peak amplitudes (n = 3). Click here for file

Additional file 2 **Detection of *Wnt-5a *Ligand under action of Aβ oligomers.** Neurons 15 DIV of culture were treated with Aβ oligomers for an hour and were immunostained for MAP2 protein and *Wnt-5a *ligand. (A), Show representative image to MAP, a: control neurons, b: Neurons treated with Aβ oligomers 50 nM, c: Neuron treated with Aβ 500 nM. Neuron was inmunostain with a specific antibody against *Wnt-5a *ligand, .d: Control Neurons, e: Neurons treated with Aβ oligomers 50 nM, f: Neuron treated with Aβ 500 nM, g: The graphs show fluorescence intensity for *Wnt-5a *ligand on neuron. Results are the mean ± S.E.M, in duplicate experiments, n = 3 separate experiments. Student's t-test *p < 0.050. (B), The soluble *Wnt-5a *ligand was detected in culture media for Hippocampal neurons 15 DIV by Sandwich ELISA technique under effect of Aβ oligomers. Click here for file

Additional file 3 **(A), Representative immunoblot of total synapsin-1 levels of neurons exposed to Aβ oligomers in the presence or absence of *Wnt-5a *and a quantification of densitometry are shown (n = 3).** (B), Representative immunoblot of total HA-Wnt-5a levels of HEK-293 cells producing conditional medium. Click here for file
