# Supplementary figures and images for: Wnt-5a occludes Aβ oligomer-induced depression of glutamatergic transmission in hippocampal neurons
Source: Mol Neurodegener. 2010 Jan 18;5:3. doi: 10.1186/1750-1326-5-3 (PMC2823745; doi:10.1186/1750-1326-5-3)

## Slide 1
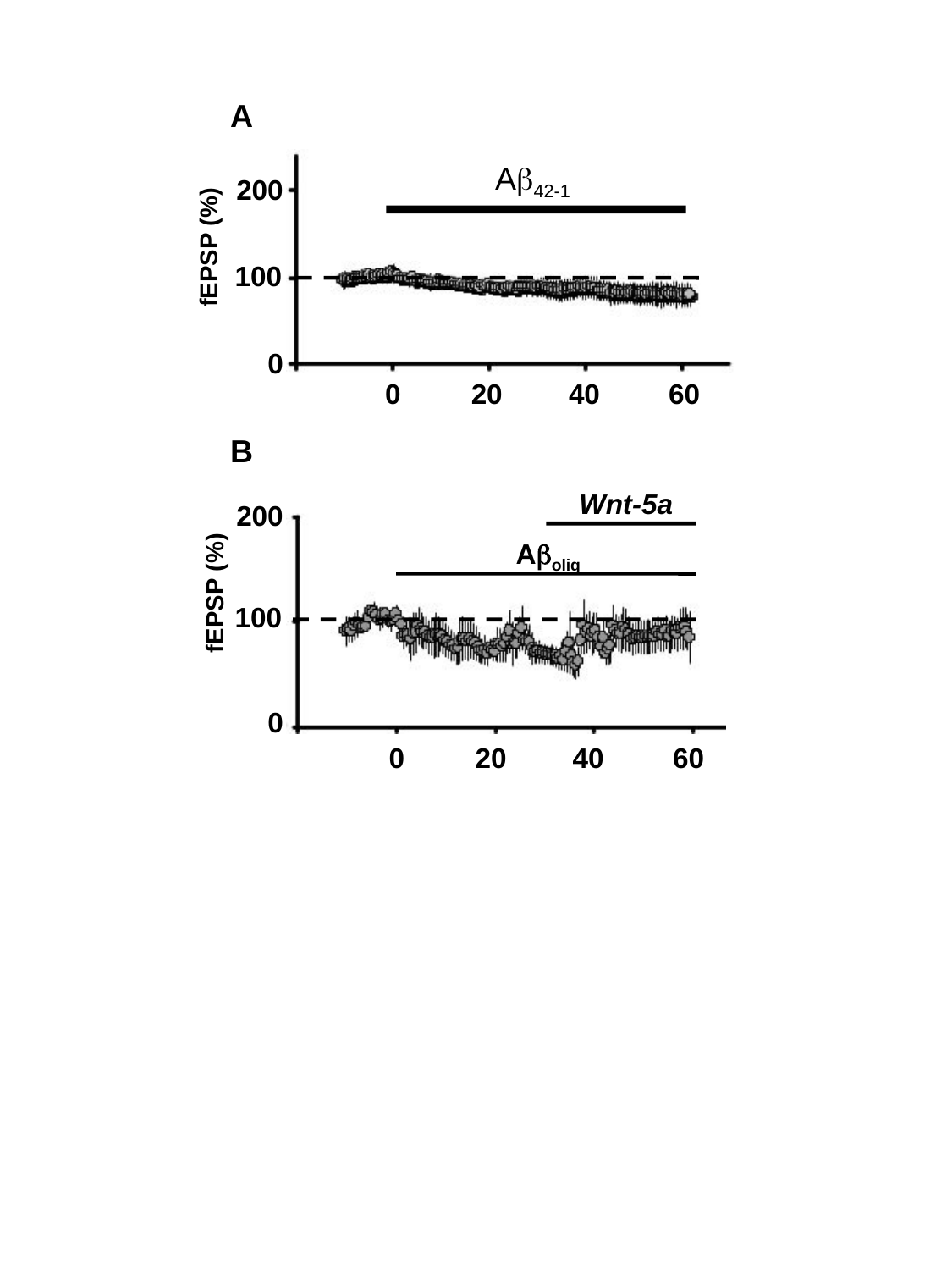

A
Aβ42-1
200
fEPSP (%)
100
0
0
20
40
60
B
Wnt-5a
200
Aβolig
fEPSP (%)
100
0
0
20
40
60

Supplement: Additional file 1 — (A), Time course of effect of Aβ42-1 (gray circles) on fEPSPs peak amplitudes (n = 4). (B), Time course of effect of Aβ oligomers and Wnt-5a after (gray circles) on fEPSPs peak amplitudes (n = 3). [file 1750-1326-5-3-S1.PPT]

## Slide 1
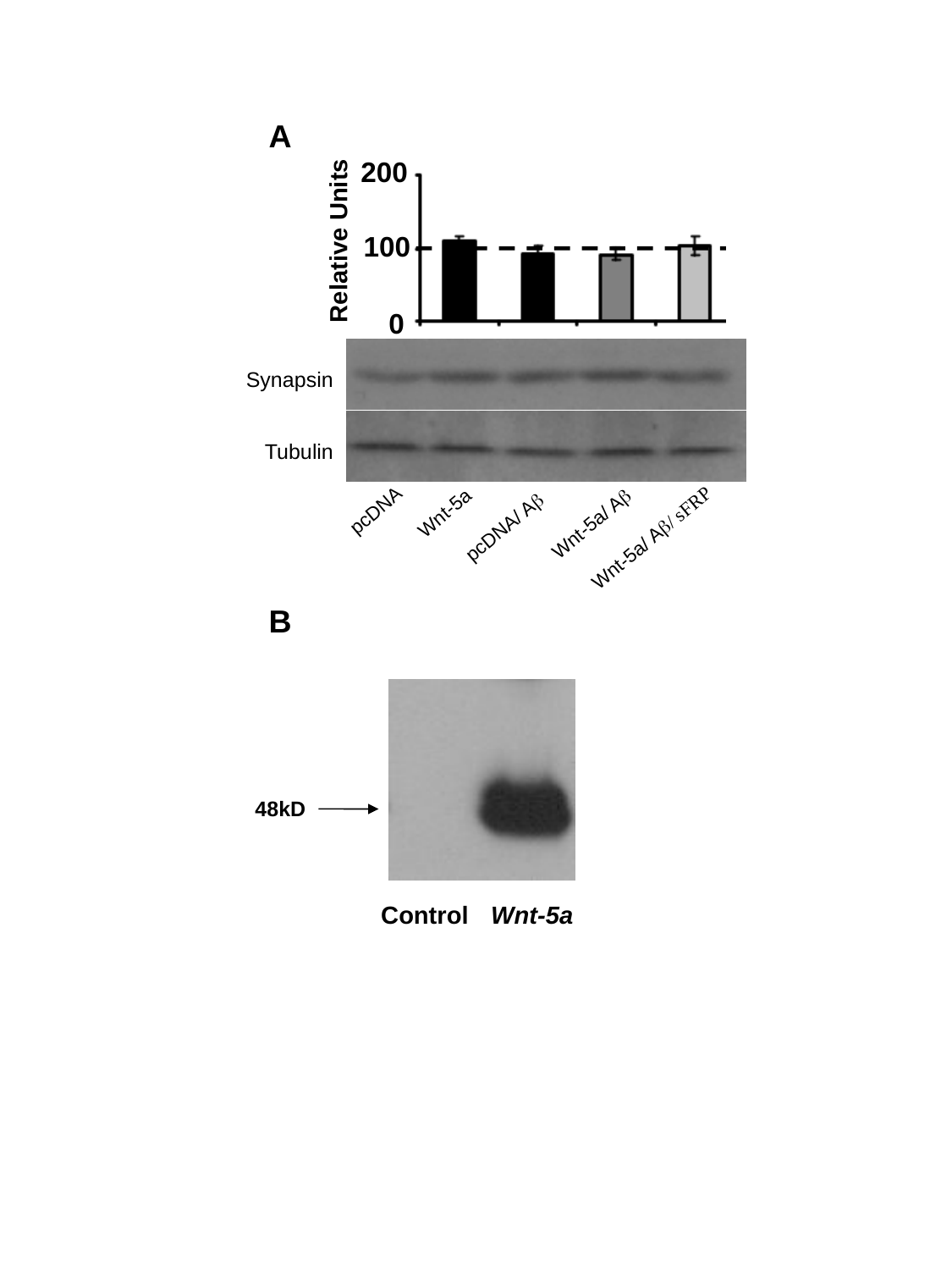

A
200
Relative Units
100
0
Synapsin
Tubulin
pcDNA
Wnt-5a
Wnt-5a/ Aβ
pcDNA/ Aβ
Wnt-5a/ Aβ/ sFRP
B
48kD
Control
Wnt-5a

Supplement: Additional file 3 — (A), Representative immunoblot of total synapsin-1 levels of neurons exposed to Aβ oligomers in the presence or absence of Wnt-5a and a quantification of densitometry are shown (n = 3). (B), Representative immunoblot of total HA-Wnt-5a levels of HEK-293 cells producing conditional medium. [file 1750-1326-5-3-S3.PPT]
