# Supplementary material for: Wnt-5a occludes Aβ oligomer-induced depression of glutamatergic transmission in hippocampal neurons
Source: Mol Neurodegener. 2010 Jan 18;5:3. doi: 10.1186/1750-1326-5-3 (PMC2823745; doi:10.1186/1750-1326-5-3)
Supplement: Additional file 2 — Detection of Wnt-5a Ligand under action of Aβ oligomers. Neurons 15 DIV of culture were treated with Aβ oligomers for an hour and were immunostained for MAP2 protein and Wnt-5a ligand. (A), Show representative image to MAP, a: control neurons, b: Neurons treated with Aβ oligomers 50 nM, c: Neuron treated with Aβ 500 nM. Neuron was inmunostain with a specific antibody against Wnt-5a ligand, .d: Control Neurons, e: Neurons treated with Aβ oligomers 50 nM, f: Neuron treated with Aβ 500 nM, g: The graphs show fluorescence intensity for Wnt-5a ligand on neuron. Results are the mean ± S.E.M, in duplicate experiments, n = 3 separate experiments. Student's t-test *p < 0.050. (B), The soluble Wnt-5a ligand was detected in culture media for Hippocampal neurons 15 DIV by Sandwich ELISA technique under effect of Aβ oligomers. [file 1750-1326-5-3-S2.PPT]

## Slide 1
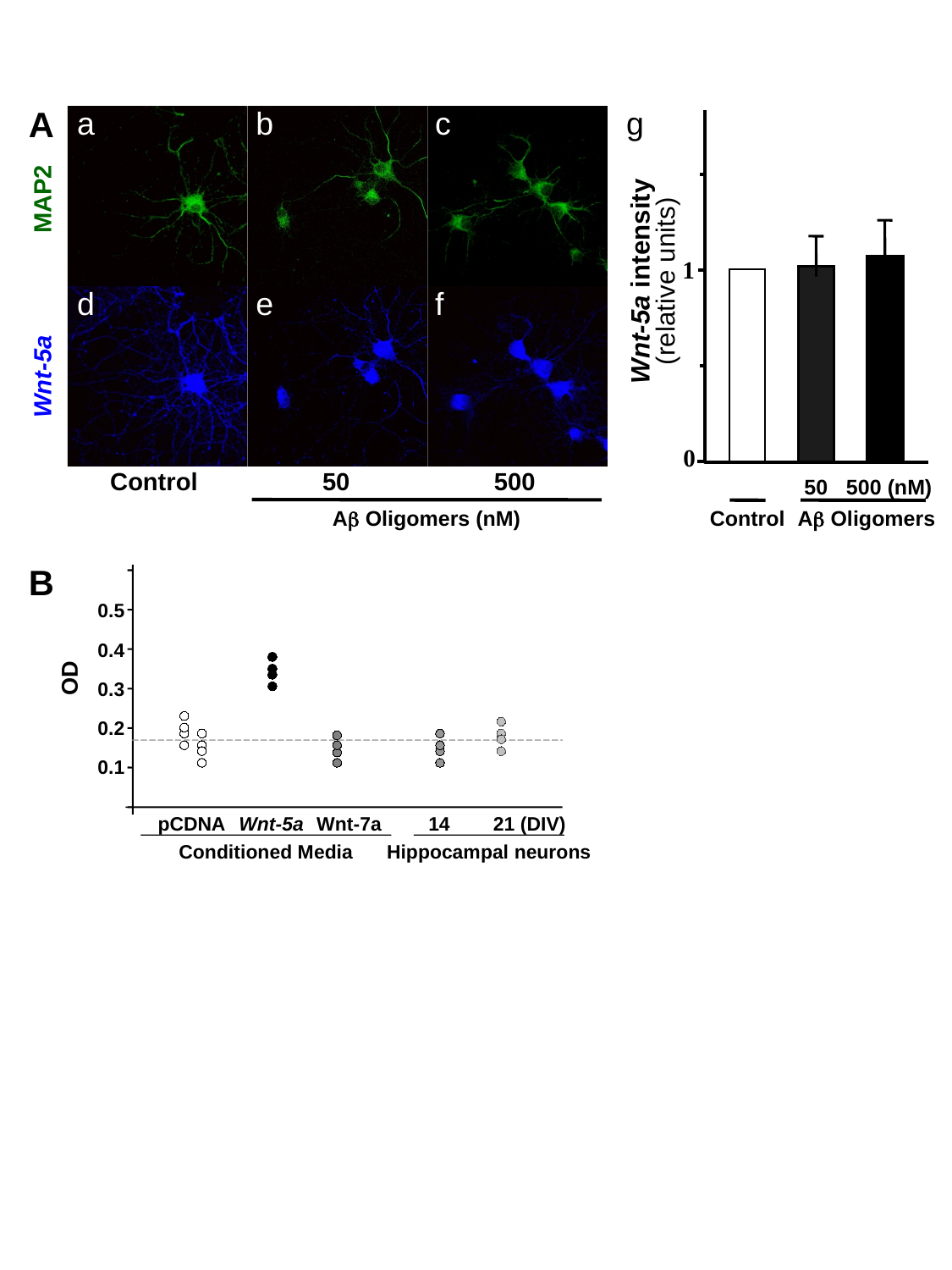

A
a
b
c
g
MAP2
1
d
e
f
 Wnt-5a intensity
 (relative units)
Wnt-5a
0
Control
50
500
50
500 (nM)
Aβ Oligomers (nM)
Control
Aβ Oligomers
B
0.5
0.4
OD
0.3
0.2
0.1
pCDNA
Wnt-5a
Wnt-7a
14
21 (DIV)
Conditioned Media
Hippocampal neurons
